# Antibodies Against Phosphorylcholine in Prediction of Cardiovascular Disease Among Women: A Population-Based Prospective Cohort Study

**DOI:** 10.1016/j.jacadv.2024.101298

**Published:** 2024-10-02

**Authors:** Johan Frostegård, Agneta Åkesson, Emilie Helte, Fredrik Söderlund, Jun Su, Xiang Hua, Susanne Rautiainen, Alicja Wolk

**Affiliations:** Units of Immunology and chronic disease, and Cardiovascular and Nutritional Epidemiology, Institute of Environmental Medicine, Karolinska Institutet, Stockholm, Sweden

**Keywords:** antibodies, cardiovascular disease, myocardial infarction, phosphorylcholine, stroke

## Abstract

**Background:**

Antibodies against phosphorylcholine (anti-PC) have been reported as associated with protection against atherosclerosis, cardiovascular disease (CVD), and other chronic inflammatory diseases. Underlying potential mechanisms have been demonstrated and include anti-inflammatory, clearance of dead cells, and inhibition of oxidized low-density lipoprotein effects.

**Objectives:**

This study examined the role of IgM anti-PC and incident CVD among women, where less is known than among men in the general population.

**Methods:**

In a total of 932 women, age 66 ± 6 years at baseline, from the population-based Swedish Mammography Cohort, IgM anti-PC levels of sera were measured using Enzyme Linked Immunosorbent assay. Prospective associations with any first CVD, ischemic heart disease (IHD), myocardial infarction (MI), and ischemic stroke were assessed using Cox proportional hazard regression, generating HRs and 95% CIs. The model was adjusted for potential confounding factors.

**Results:**

Over the course of 16 years (13,033 person-years), we identified 113 cases of composite CVD, 69 cases of IHD, 44 cases of MI, and 50 cases of ischemic stroke. IgM anti-PC was statistically significantly inversely associated with risk of CVD, IHD, and MI, but not with ischemic stroke. Comparing the highest tertile with lowest, we observed multivariable-adjusted HR of 0.27 (95% CI: 0.11-0.68; *P* trend <0.01) for MI.

**Conclusions:**

IgM anti-PC may play an active role in inhibition of CVD development in women, especially MI. Furthermore, IgM anti-PC levels may play a role in identifying those at risk.

Atherosclerosis is an inflammatory condition located mainly in the intima of arteries forming atherosclerotic plaques, damage of which is of major importance in etiology of cardiovascular disease (CVD). Typical of atherosclerosis is the accumulation of dead cells forming a necrotic core, many originating from foam cells, which are inert macrophages filled with lipids such as oxidized low-density lipoprotein (OxLDL). OxLDL has proinflammatory and immune stimulatory properties. Typical of plaques is also the presence of activated immune competent cells in the lesions, producing mainly proinflammatory cytokines.^1^^,^^2^

Phosphorylcholine (PC) is a small hapten, which is abundant in cell membranes and lipoproteins and IgM antibodies against PC (anti-PC) are present in the circulation, at relatively high levels in humans, but only recognize PC when PC is exposed, as in dead cells and OxLDL. PC is reported to be central to oxLDL-induced immune activation contributing to its proinflammatory effects.^3^ PC is also a pathogen-associated molecular pattern, exposed on bacteria including *Streptococcus pneumoniae*^1^^,^^4^ and other microorganisms as nematodes and parasites, which is relevant in the context of CVD too.^1^^,^^5^^,^^6^

Common underlying risk factors for CVD which have been discussed include established risk factors for CVD-like smoking, dyslipidemia, diabetes type 2 and hypertension, but also oxidative stress and dietary factors. In addition, male gender and age are associated with increased risk.^1^ Chronic low-grade inflammation and atherosclerosis are other features of CVD, which may be consequences of inflammatory aging.^7^

We previously reported that IgM anti-PC could be a protection marker in CVD including both stroke and myocardial infarction (MI), atherosclerosis progress, and also mortality after MI,^1^^,^^6^^,^^8^^,^^9^ as well as in other chronic inflammatory and/or autoimmune conditions, including systemic lupus erythematosus (SLE) and rheumatoid arthritis.^1^^,^^10^, ^11^, ^12^ These findings have largely been confirmed and also extended into other diseases like vasculitis and osteoarthritis.^13^, ^14^, ^15^, ^16^, ^17^, ^18^, ^19^, ^20^

Based on our hypothesis that there are inverse associations between CVD and IgM anti-PC, low levels leading to high risk, and high levels leading to low risk, we studied IgM anti-PC in a prospective cohort of women, since studies so far have had more men included.^6^ We explored, for the first time among women, the association between serum IgM anti-PC and composite CVD, and specifically ischemic heart disease (IHD), MI, and ischemic stroke. We hypothesize that anti-PC at high levels is associated with lower risk, especially against MI. The implications are discussed.

## Materials and methods

### Study population

**Figure 1:**
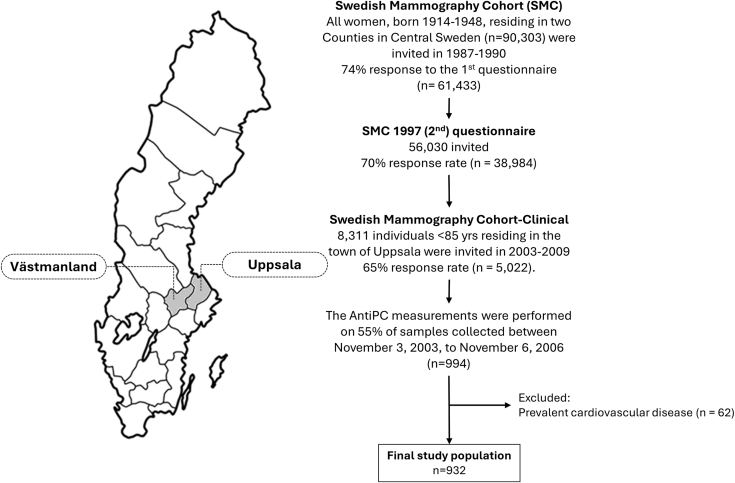
Flow Diagram of the Study Population

The Swedish Mammography Cohort is a population-based prospective cohort,^21^ and a part of the Swedish Infrastructure for Medical Population-based Life-Course and Environmental Research (SIMPLER). It was established in 1987 to 1990, when all women born 1914 to 1948 in Uppsala County and 1917 to 1948 in Västmanland County (n = 90,303) were sent a questionnaire on diet and lifestyle (response rate 74%). From 2003 through 2009, a clinical subcohort was established (The Swedish Mammography Cohort-Clinical), setting the baseline for the current investigation. Herein, 8,311 women residing in Uppsala age <85 years were reinvited for a health examination. Enclosed with the invitation was an updated questionnaire on diet and lifestyle factors. In total, 65% of the invited women returned a completed questionnaire and 61% took part in the health examination. The examination included collection of blood samples, measurements of blood pressure, weight, height, waist, hip, and a dual-energy x-ray absorptiometry scan. Venous blood samples were collected after a 12-hour overnight fast and samples were immediately centrifuged and separated in a dark room and stored at −80 °C until analysis. For this study, we included 55% (n = 994) of the women that provided blood samples between November 3, 2003 and November 6, 2006. These women were selected based on overlap with other biomarkers randomly measured. The 55% of the women included in this study were slightly younger (mean age 67 ± 6 years vs 70 ± 7 years) but did not differ compared to those without anti-PC measurement with respect to body mass index (BMI) and LDL cholesterol. From the 994 women with measurements of serum anti-PC, we then excluded prevalent cases of ischemic stroke and IHD, resulting in 932 participants representing the final study population of the present investigation (Figure 1). Written informed consent was obtained from all participants and ethical permission was approved by the Regional Ethical Review Board in Stockholm, Sweden.

### Exposure assessment and covariates

#### Assessment of anti-PC

IgM anti-PC concentrations of sera from individuals in the study were measured with an indirect noncompetitive enzyme immunoassay (CVDefine, Athera Biotechnologies AB) according to the manufacturer's instructions, as described previously. Briefly, the kit is based on PC being covalently linked to bovine serum albumin coated onto 96-well Nunc Maxisorp microtiter plates. All readings of results were performed on Enzyme Linked Immunosorbent assay Multiscan Plus spectrophotometer (Molecular Devices Emax).^22^

#### Assessment of covariates

Self-reported information on education level, smoking status, and alcohol consumption was obtained from the baseline questionnaire, November 2003 to September 2009, while ever use of corticosteroids and aspirin, and family history of MI in parents or siblings before age 60 years was obtained from the preceding questionnaire (1997). BMI was calculated from weight and length obtained at the clinical examination, and total and LDL cholesterol were measured in the blood samples. Participants with total cholesterol ≥5.2 mmol/L and LDL cholesterol ≥4.1 mmol/L were classified as prevalent hypercholesterolemia.^23^^,^^24^ Information on prevalent diabetes and hypertension was based on linkage to the National Patient Register and on self-reported data from the questionnaire.

### Outcome assessment

Information about incident disease was obtained via a computerized linkage to the National Patient Register using the International Classification of Diseases-10th Revision codes. The International Classification of Diseases-10th Revision codes for IHD were I20-I25; for MI: I21; and for ischemic stroke: I63.

### Statistical analysis

Since there is not yet predefined IgM anti-PC cutoff, participants were categorized into quintiles of baseline serum IgM anti-PC to assess the association of composite CVD and IHD. Because of the limited number of MI and ischemic stroke cases, we applied tertiles in the assessment of those 2 outcomes. Associations with any incident CVD, IHD, MI, and ischemic stroke were assessed using Cox proportional hazard regression with attained age as underlying time scale, generating HRs and 95% CIs. Participants contributed with person-time from the date of examination until the date of event under evaluation, death, or end of follow-up (December 31, 2020), whichever came first. Test for linear trend (*P* trend) was conducted by assigning each participant the median value of serum anti-PC in each quantile, and modeled as a continuous variable. To flexibly model the dose-response associations and allow for nonlinearity, we used restricted cubic splines with 4 knots (at the 5th, 35th, 65th, and 95th percentiles of the distribution), excluding outliers corresponding to >99th percentile (anti-PC >289 U/mL).

The model was adjusted for potential confounding factors, a priori selected based on being associated with inflammation as well as being known risk factors for the outcomes: age (continuous), education (≤9, 9-12 and >12 years), BMI (continuous), smoking (never, former, current), alcohol (≤0.5, 0.5-15, >15 g/day), ever use of corticosteroids (yes/no), use of aspirin (yes/no), family history of MI (yes/no), prevalent diabetes at baseline (yes/no), prevalent hypertension at baseline (yes/no), prevalent hypercholesterolemia at baseline (yes/no). Missing information on covariates (<2%) was handled by placing missing values for categorical variables in a separate category, while missing variables for continuous variables received the median value for the respective variable. Nonresponders for use of corticosteroids (27%) and aspirin (11%) were regarded as nonusers.

The statistical analyses were performed using STATA/BE version 17 (Stata Corporation, Inc) where each test was 2-sided with a significance level of 0.05.

## Results

The participants’ mean age was 66 ± 6 years. The mean serum IgM anti-PC at baseline was 70 ± 66 U/mL and the median was 53 (5th-95th percentile 16-165) U/mL. Baseline age-standardized characteristics of the 932 women by quintiles of serum IgM anti-PC are shown in Table 1. No major differences were observed across baseline quintiles other than a higher proportion of current and former smokers and lower prevalence of hypertension in the highest quintile.

**Table 1:** Baseline Age-Standardized Characteristics in 932 Women by Baseline Quintiles of IgM Anti-PC Concentrations

	Quintiles of Serum Anti-PC				
	1 (n = 187)	2 (n = 186)	3 (n = 187)	4 (n = 186)	5 (n = 186)
Mean anti-PC (U/mL)	20 ± 6	36 ± 4	53 ± 6	81 ± 10	161 ± 96
Median anti-PC (U/mL)	20 (10-28)	36 (29-42)	53 (44-63)	80 (65-98)	134 (105-289)
Age, y	67 ± 6	66 ± 6	67 ± 6	65 ± 6	66 ± 6
Education >12 y	43	42	46	39	35
BMI, kg/m^2^	27 ± 5	26 ± 4	26 ± 4	26 ± 4	26 ± 4
Smoking status					
Never	62	61	62	49	45
Former	27	30	31	35	43
Current	10	8	7	17	12
Alcohol, g/d					
≤0.5	22	18	19	25	28
0.5–15	72	75	77	70	65
>15	6	7	4	4	7
Ever use of corticosteroids	7	6	6	5	6
Ever use of aspirin	50	44	47	42	47
Family history of myocardial infarction	16	12	16	10	17
Prevalent diabetes	7	5	3	5	8
Prevalent hypertension	12	7	8	3	9
Hypercholesterolemia^a^	31	33	32	26	29

anti-PC = antibodies against phosphorylcholine.

Values are mean ± SD, median (5th-95th percentile), or %.

a ≥5.2 mmol/L for total cholesterol and ≥4.1 mmol/L for low-density cholesterol.

**Figure 2:**
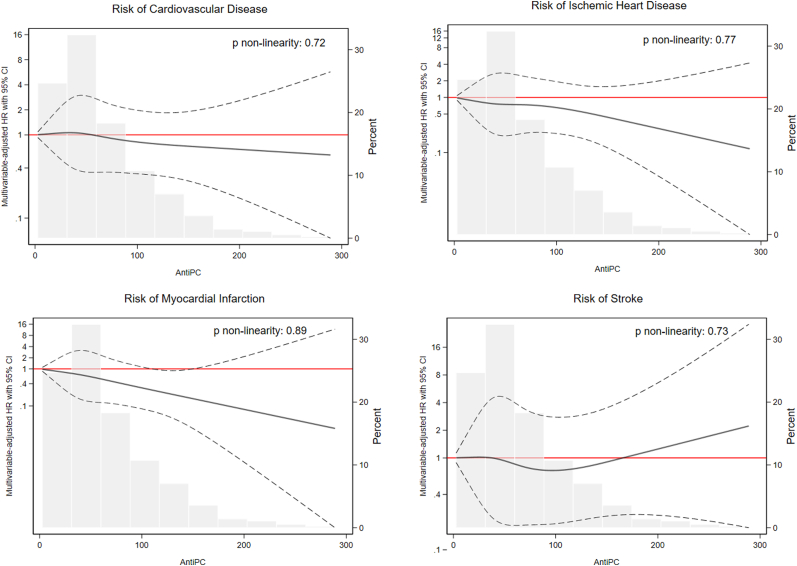
**HRs With 95% CIs of Cardiovascular Disease, Ischemic Heart Disease, Myocardial Infarction, and Stroke as a Function anti-PC (U/mL), Evaluated Using Restricted Cubic Splines With 4 Knots (Corresponding to 5th, 35th, 65th, and 95th Percentiles of the Distribution) Excluding Outliers >99th Percentile** The histograms show the distributions of the anti-PC concentrations. Models were adjusted for attained age (years), education, BMI, smoking, alcohol intake, steroid use, aspirin use, family history of myocardial infarction, prevalence of diabetes, prevalence of hypertension, and prevalence of hypercholesterolemia. Solid lines = HRs; dashed lines = 95% CIs; anti-PC = antibodies against phosphorylcholine.

Over the course of 16 years (13,033 person-years), we ascertained 113 cases of composite CVD, 69 cases of IHD, 44 cases of MI, and 50 cases of ischemic stroke. As shown in Table 2, we observed significant associations with lower risk of composite CVD and IHD comparing highest quintile of IgM anti-PC with lowest; multivariable-adjusted HRs: 0.42 (95% CI: 0.21-0.84; *P* trend = 0.06) for composite CVD and 0.35 (95% CI: 0.14-0.84; *P* trend = 0.05) for IHD. Comparing the corresponding tertiles, HRs for MI and ischemic stroke were 0.27 (95% CI: 0.11-0.68; *P* trend <0.01) and 0.82 (95% CI: 0.40-1.67; *P* trend 0.61), respectively (Table 3). Restricted cubic spline analyses (Figure 2) indicated no deviation from linearity (*P* nonlinearity ≥0.72) (Figure 2).

**Table 2:** HRs (95% CI) Estimating the Associations Between Quintiles of IgM Anti-PC Concentrations at Baseline and Incidence of Composite Cardiovascular Disease and Ischemic Heart Disease

	Quintiles of Serum Anti-PC					
	1	2	3	4	5	*P* Trend
Anti-PC (U/mL)	20 ± 6	36 ± 4	53 ± 6	81 ± 10	161 ± 96	
Composite CVD						
Number of cases	30	18	27	26	12	
Person-years	2,473	2,717	2,567	2,639	2,638	
Age-adjusted HR (95% CI)	1.00 (reference)	0.56 (0.31-1.00)	0.88 (0.52-1.48)	0.89 (0.53-1.51)	0.40 (0.21-0.79)	0.05
Multivariable-adjusted^a^ HR (95% CI)	1.00 (reference)	0.62 (0.35-1.13)	1.02 (0.60-1.74)	1.06 (0.61-1.84)	0.42 (0.21-0.84)	0.06
Ischemic heart disease						
Number of cases	20	11	17	14	7	
Person-years	2,517	2,763	2,644	2,702	2,673	
Age-adjusted HR (95% CI)	1.00 (reference)	0.51 (0.25-1.07)	0.81 (0.42-1.54)	0.69 (0.35-1.38)	0.34 (0.14-0.80)	0.04
Multivariable-adjusted^a^ HR (95% CI)	1.00 (reference)	0.59 (0.28-1.23)	0.94 (0.48-1.81)	0.88 (0.43-1.80)	0.35 (0.14-0.84)	0.05

Abbreviation as in Table 1.

a Multivariable-adjusted models were adjusted for age, education, BMI, smoking, alcohol intake, steroid use, aspirin use, family history of myocardial infarction, prevalence of diabetes, prevalence of hypertension, and prevalence of hypercholesterolemia.

**Table 3:** HRs (95% CI) Estimating the Associations Between Tertiles of IgM Anti-PC Concentrations at Baseline and Incident Myocardial Infarction and Ischemic Stroke

	Tertiles of Serum Anti-PC			
	1	2	3	*P* Trend
Anti-PC (U/mL)	25 ± 8	54 ± 10	131 ± 83	
Myocardial infarction				
Number of cases	21	17	6	
Person-years	4,391	4,537	4,571	
Age-adjusted HR (95% CI)	1 (Ref.)	0.80 (0.42, 1.52)	0.30 (0.12, 0.75)	
Multivariable-adjusted^a^ HR (95% CI)	1 (Ref.)	0.80 (0.41, 1.53)	0.27 (0.11, 0.68)	0.005
Ischemic stroke				
Number of cases	20	16	14	
Person-years	4,432	4,512	4,525	
Age-adjusted HR (95% CI)	1 (Ref.)	0.84 (0.43, 1.62)	0.81 (0.41, 1.62)	
Multivariable-adjusted^a^ HR (95% CI)	1 (Ref.)	0.86 (0.44, 1.67)	0.82 (0.40, 1.67)	0.61

Abbreviation as in Table 1.

a Multivariable-adjusted models were adjusted for age, education, BMI, smoking, alcohol intake, steroid use, aspirin use, family history of myocardial infarction, prevalence of diabetes, prevalence of hypertension, and prevalence of hypercholesterolemia.

## Discussion

In this population-based study among postmenopausal women, we observed that IgM anti-PC may be a marker of protection against incident composite CVD, mainly for IHD and MI, with the latter corresponding to a multivariable-adjusted 73% (95% CI: 32%-89%) lower risk in the highest compared to lowest tertile. The inverse associations between IgM anti-PC and development of ischemic stroke during follow-up did not reach statistical significance (Central Illustration).

**Central Illustration:**
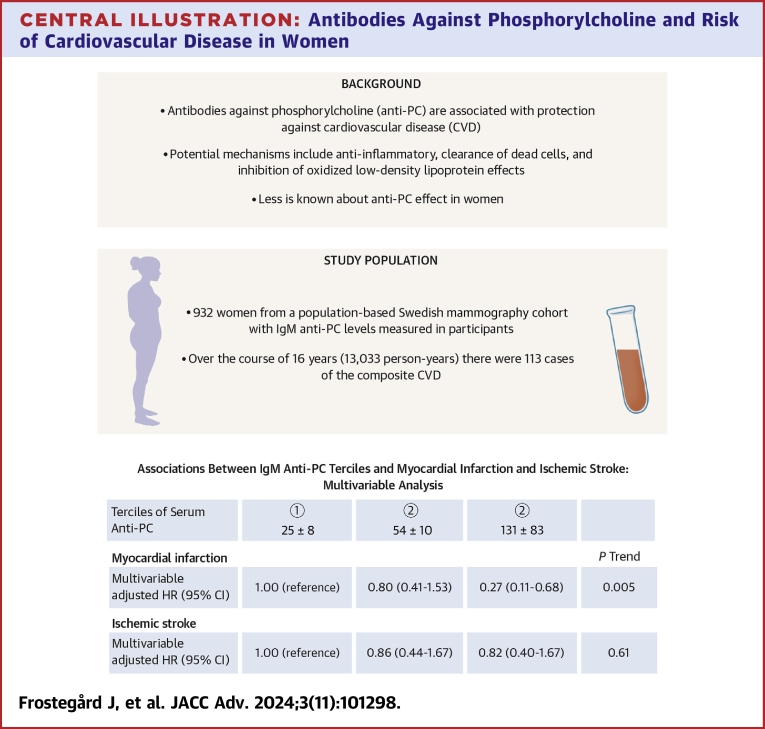
Antibodies Against Phosphorylcholine and Risk of Cardiovascular Disease in Women

Previous CVD studies based on a nested case-control design were dominated by male subjects, or had outcomes of specific diseases in focus.^6^ Our findings are the first based on prospective study where women are in focus and results are in line with previous studies,^1^^,^^6^^,^^8^, ^9^, ^10^, ^11^, ^12^^,^^15^^,^^17^^,^^19^^,^^25^, ^26^, ^27^, ^28^, ^29^ also expanded into chronic inflammatory conditions.^9^^,^^13^, ^14^, ^15^, ^16^, ^17^, ^18^^,^^20^^,^^26^, ^27^, ^28^, ^29^ This is of interest since those conditions commonly lead to increased risk of CVD.^1^ These findings have been expanded to other subclasses and isotypes of anti-PC including IgG, IgG1, and IgG2 anti-PC and similar association with protection was determined for IgG1 anti-PC.^6^ Initially, anti-PC among hypertensives was protectively associated with atherosclerosis progression after 4 years.^9^ In autoimmune conditions, it was reported in a population-based prospective cohort of patients with rheumatoid arthritis that the risk of incident CVD is inversely associated with IgM anti-PC both among men and women, using the same Enzyme Linked Immunosorbent assay method as herein.^30^ In another prospective study on uremic patients on dialysis, we demonstrated significant inverse associations with mortality.^31^

Future studies should define cutoff levels for IgM anti-PC. Potentially this cutoff is sex-specific, as IgM anti-PC is higher among women than among men in all studies we are aware of, both in health and disease.^6^ The reason for the sex differences could only be speculated, but appears to have an evolutionary background, since female individuals leading a traditional life as hunters, gatherers, and horticulturalists also have higher levels than their male counterparts.^32^^,^^33^ This sex difference agrees well with the older age of first CVD in women as compared to men, since higher levels of anti-PC could increase protection against atherosclerosis and chronic inflammation in general. Interestingly, in rheumatic diseases, which mostly affect women, IgM anti-PC is inversely associated with CVD, in spite of women having higher levels than men. Thus, women appear to benefit from having even higher anti-PC levels. Still, the findings may or may not be generalizable to women of all races and ethnicities.

In atherosclerotic lesions, several damage-associated molecular patterns (DAMPs) are expressed on damaged and dead cells and also oxLDL. These have as a function to attract and activate the body’s defense systems and can be proinflammatory or even dampen inflammation, depending on context. Examples of DAMPs include malondialdehyde and PC, among others. Both have proinflammatory properties, when exposed on OxLDL and in other contexts (though not on apoptotic cells), and may thus contribute to OxLDL’s immune stimulatory and proinflammatory properties.^3^^,^^34^^,^^35^ Also compounds as cardiolipin, especially oxidized cardiolipin and phosphatidylserine can function as DAMPs.^1^

PC is abundant as a polar head group in membrane phospholipids.^36^ Phosphatidylcholine in cells and LDL can be modified by oxidation, chemically, or through enzymes such as phospholipase A2 when it loses fatty acids in the sn-2 position, exposing PC. This oxidized compound also has proinflammatory effects and promotes atherosclerosis in mouse models.^36^^,^^37^ In addition to being a DAMP, PC, in contrast to the other mentioned DAMPs, is also a pathogen-associated molecular pattern. It has been known since long that PC is exposed in the cell wall of *S pneumoniae* and immunization with PC can ameliorate bacterial infection in animal models.^38^, ^39^, ^40^, ^41^ PC is also well known as an antigen exposed in other microorganisms including nematodes and helminths.^42^, ^43^, ^44^

There are several potential mechanisms described in experimental systems, which lend support to the notion that IgM anti-PC is not only associated with MI, other types of CVD, atherosclerosis, and other chronic inflammatory disease conditions, but could play a causative role. Anti-PC has anti-inflammatory properties, inhibiting the effects of inflammatory phospholipids, which depend on PC exposed, typically in platelet-activating factor and platelet-activating factor-like lipids forming during LDL-oxidation.^11^ Another property of anti-PC is to inhibit OxLDL uptake into macrophages and inhibition of foam cell formation could be implicated as a protective mechanism.^22^ Furthermore, human IgM anti-PC is clearance of dead cells.^45^ This could be an important factor in atherosclerosis and also in autoimmune conditions like SLE where the prevalence of atherosclerotic plaques and risk of CVD is high. In atherosclerosis, the disease process itself is characterized by the accumulation of dead cells in the artery wall of affected vessels and in SLE, clearance of apoptotic cells is known to be abberrant.^1^^,^^2^^,^^11^^,^^45^

Another mechanism which could be protective in chronic inflammatory conditions is that IgM anti-PC promotes polarization of T regulatory cells (Tregs), both from atherosclerotic plaques and blood of patients with SLE.^46^ Both animal experiments^47^ and human studies^48^ indicate that Tregs are implicated as protective factors in atherosclerosis and thus CVD. In SLE, Tregs were lower than among controls which are in line with previous studies where those cells were negatively associated with odds of SLE.^49^ Recently, IgM anti-PC was confirmed to be lower in SLE as compared to age- and sex-matched controls and associated with the prevalence of atherosclerotic plaques in this condition. Moreover, the levels of Tregs were normalized by IgM anti-PC addition to cell cultures and were raised to the same levels as in healthy controls.^46^ Also, animal models support the notion that anti-PC has atheroprotective properties. PC immunization, raising anti-PC levels 3-fold, reduced atherosclerotic aorta root lesions by >40% in an apolipoprotein E k/o mouse model (*P* < 0.01). Also, foam cell formation was reduced.^50^

Little is known about the development of IgM anti-PC over time in the same individuals, while there is a negative association with age in a Western population, this was not observed in a group of hunter-gatherers and horticulturalists, with a favorable risk factor profile, with low blood pressure and high level of mobility and low BMI and high IgM anti-PC. It can be hypothesized that low exposure to infectious agents exposing PC in Western countries could lead to an immune deficient state with less protection against these chronic inflammatory conditions (and infections).^32^^,^^33^

### Study Limitations

The limitation of this study is the sample size with restricted number of incident cases. Clearly, larger prospective population cohort studies are needed to further explore the associations. In addition, given the observational design, we cannot exclude the possibility of unmeasured or some residual confounding, also in relation to binary variables. Strengths are the population-based design, detailed information on several covariates either self-reported or measured at the health examination. The linkage to high-quality national registers enabling an almost complete ascertainment of cases is also an important asset.

## Conclusions

We observed that IgM anti-PC is inversely and statistically significantly associated with incident IHD and MI. IgM anti-PC may thus represent a risk marker in these disease conditions. The possibility that raising anti-PC through immunization in order to prevent and/or delay IHD deserves further investigations.

PERSPECTIVES **COMPETENCY IN MEDICAL KNOWLEDGE:** CVD is a major cause of disease and death among men and women. The role of chronic inflammation in atherosclerosis, which is a major cause of CVD, has been discussed for long. Still, the possibilities to predict the role of inflammation in CVD are not strong, even though C-reactive protein has been implicated. Women have higher life expectancy than men, and one reason is later onset of CVD. We here report that anti-PC is a strong predictor of CVD among women, low levels associated with increased risk. This adds to previous knowledge of the role of anti-PC and may improve prediction and also prevention.

**TRANSLATIONAL OUTLOOK:** We have provided a novel hypothesis about the development of the chronic inflammation leading to atherosclerosis and CVD, namely that low levels of anti-PC, among men and also women, contribute. Mechanisms include anti-inflammatory and increased clearance of dead cells and oxidized lipids. The possibility to raise anti-PC, eg, through immunization, therefore deserves further studies.

## Funding support and author disclosures

This work was supported by the Swedish Heart and Lung Foundation, Swedish Science Fund, King Gustav V 80-year fund, CVDIMMUNE (EU). The authors have reported that they have no relationships relevant to the contents of this paper to disclose except that Johan Frostegård is named as inventor on other aspects of anti-PC than associations with any cardiovascular disease studied herein.
